# Evolutionary dynamics and research hotspots of phage applications against *Acinetobacter baumannii* infections from the past to the new era

**DOI:** 10.3389/fmicb.2025.1606351

**Published:** 2025-06-24

**Authors:** Ping Jiang, Xiaoqin Luo, Jing Zhao, Jingwei Sun, Zhanhai Su, Peng Cheng

**Affiliations:** ^1^Department of Basic Medical Sciences, Qinghai University Medical College, Xining, China; ^2^Department of Clinical Laboratory, Qinghai Provincial People’s Hospital, Xining, China; ^3^Research Center for High Altitude Medicine, Key Laboratory for High Altitude Medicine, Ministry of Education, Qinghai University, Xining, China

**Keywords:** *Acinetobacter baumannii*, phages therapy, bibliometric analysis, biofilm, phage resistance, synergistic effect

## Abstract

**Background:**

*Acinetobacter baumannii* is a common hospital pathogen that poses a serious clinical challenge due to its rapidly increasing resistance to antibiotics. Phage therapy has been successfully used to treat antibiotic resistant *A. baumannii* infections. The aim of this study was to comprehensively assess the current status and trend of research on the application of phages in *A. baumannii* infections through bibliometric analysis.

**Methods:**

Studies on phages and *A. baumannii* infections were searched in the Web of Science Core Collection database and relevant articles were selected for inclusion in the study based on the inclusion criteria. Bibliometric and visual analysis of the included publications were performed using VOSviewer and CiteSpace software.

**Results:**

A total of 264 studies were included. There is an increasing trend in the number of publications per year from 2010 to 2024. China was the leading country with 35.98% of the total publications. Tzu Chi University and Lin, Nien-Tsung were the most influential institution and author, respectively. The journal with the highest H-index was Frontiers in Microbiology, and Viruses-Basel was the most prolific journal. Antimicrobial Agents and Chemotherapy was the most cited journal. Phages endolysin and phage therapy were found to be the widely researched aspects, biofilm, phage resistance and synergistic effect are recent research hotspots.

**Conclusion:**

In the last decade or so, this is the first bibliometric study that systematically describes the research hotspots and development trends on phages in *A. baumannii* infections. Research hotspots should be given more attention.

## 1 Introduction

*Acinetobacter baumannii* is an important Gram-negative pathogenic bacterium that is widespread in nature (Pandey et al., 2022). It can cause a variety of infections including meningitis, endocarditis, ventilator-associated pneumonia in intensive care, urinary tract infections and bacteremia (Antunes et al., 2014; Li et al., 2023). Study showed that the six leading pathogens for deaths associated with resistance were responsible for 929,000 deaths attributable to Antimicrobial resistance (AMR) and 3.57 million deaths associated with AMR in 2019, and that *A. baumannii* is one of them (Antimicrobial Resistance Collaborators, 2022). Furthermore, *A. baumannii* has demonstrated a remarkable ability to rapidly evolve resistance to conventional antibiotics, leading to the emergence of multidrug-resistant *A. baumannii* (MDRAB), extensively drug-resistant *A. baumannii* (XDRAB) and pan-resistant *A. baumannii* (PDRAB) strains. In particular, carbapenem-resistant *A. baumannii* (CRAB) is on the World Health Organization (WHO) list of critical priority bacteria for which new antimicrobial strategies are urgently needed (Tacconelli et al., 2018). As current antibiotics are becoming ineffective and there are still some challenges in the discovery and synthesis of new antibiotics, much effort has been directed towards the development of new therapeutic options to combat AMR, including phage or phage therapeutics, phage-encoded products, etc. (Chang et al., 2022). As of today the application of phages as antimicrobial agents has received increasing attention as a promising and safe therapeutic supplement for the treatment of bacterial infections (Nale and Clokie, 2021; Satta et al., 2022).

Phages are the most abundant and ubiquitous organisms on Earth and play important roles in microbial physiology, population dynamics, evolution and therapy (Clokie et al., 2011). Specifically speaking, because they are viruses, phages can target and kill their specific host bacteria, including antibiotic-resistant strains. Phages with a forced lysis life cycle are used in phage therapy, where they inject their genetic material into host bacteria, replicate themselves and then release their progeny through lysis. Unlike antibiotics, phages have high host specificity and low intrinsic virulence, minimizing interference with normal flora (Chang et al., 2020; Chang et al., 2022; Chang et al., 2018; Chang et al., 2017). Since the early 1920s, phages have been considered as therapeutic agents due to their unique antimicrobial capacity. Phages are considered to be one of the most promising alternatives to conventional antibiotics due to their high antibacterial capacity, large number (10^30^ ~ 10^32^ in the earth), low toxicity and side effects to humans (Hibstu et al., 2022; Tu et al., 2023; Uyttebroek et al., 2022).

Phage therapy relies on the precise use of phages to attack and kill bacteria and effectively control infections (Abedon et al., 2011). Since the first report of the protective effect of phage BS46 on mice infected with *A. baumannii* strain AC54 (Soothill, 1992), more and more phages have been identified for lysing *A. baumannii*, and some phages have even shown promising antimicrobial properties in animal models (Wang et al., 2023). In addition to live phages, some phage-derived enzymes, such as intracellular lysins and depolymerases, have been shown to possess bactericidal activity (Chen et al., 2022; Chu et al., 2022). Furthermore, the efficacy and safety of phage therapy has been demonstrated (Zhang et al., 2022). Case reports of pneumonia (Cha et al., 2018), wound infections (Regeimbal et al., 2016), bacteraemia (Leshkasheli et al., 2019) and sepsis (Jasim et al., 2018), as well as phage therapy targeting patients with a variety of diseases that are carbapenem-resistant or MDRAB demonstrate the potential value of phage therapy in clinical practice (Schooley et al., 2017; Tan et al., 2021; Wu N. et al., 2021). The most common phage therapies currently used against *A. baumannii* include single phage therapies (McCallin et al., 2018), phage mixtures (Molina et al., 2021), phage-antibiotic combination therapies (Luong et al., 2020b), and novel phage therapies such as phage-derived enzymes (Oliveira et al., 2018) and photosensitizer combination therapies (Ran et al., 2020; Zhang et al., 2022). Phage therapy has also been reported as a promising alternative to address the ongoing problem of *A. baumannii* biofilm infections (Teymouri et al., 2024).

Bibliometric analysis is a statistical method based on public literature databases that allows for quantitative and qualitative evaluation of publications to assist in analyzing research hotspots and trends in specific fields (Zhang et al., 2020). To the best of our knowledge, no bibliometric analysis have been published on the use of phages in *A. baumannii* infections. In this study, we aimed to use quantitative methods to analysis the application of phages in *A. baumannii* infections, to identify the main contributors and the current state of research in this field, and to suggest future research trends.

## 2 Materials and methods

### 2.1 Data source

The data analyzed are based on the Web of Science Core Collection (WOSCC) database. We chose WOSCC as a data source because it is more selective than other databases in terms of scientific coverage (Gazzaz et al., 2020). WOSCC is considered to contain not only the most comprehensive publications and high-quality indexes (Gazzaz et al., 2020), but also complete references and citations (Mongeon and Paul-Hus, 2016), making it a more comprehensive data source and the most widely used database in bibliometric research.

### 2.2 Search strategy

All studies until February 22, 2025 were retrieved and downloaded from the WOSCC database. The search strategy to obtain articles on phages in *A. baumannii* infections involved “TS = (Phage OR Phages OR Bacteriophage) AND TS = (*Acinetobacter baumannii* OR Bacterium anitratum).”

### 2.3 Data extraction

All relevant publications were independently assessed by two authors (Ping Jiang and Xiaoqin Luo) in two stages. In the first stage of screening, the language of publications was limited to English. In addition, non-article studies (reviews, conference proceedings, letters, etc.) were excluded from our study. In the second stage, the titles and abstracts of the remaining studies were carefully assessed and selected to include only articles that focused on phages in *A. baumannii* infections, using the following criteria: P (patients): studies involving patients with *A. baumannii* infections, animal models of *A. baumannii* infections and cellular models of *A. baumannii* infections; I (interventions): application of phages; S (study design): clinical and basic research. Disagreements were resolved by discussion.

### 2.4 Data analysis

The included publications and cited references were exported in plain text and subsequently analyzed for bibliometric and visualization purposes. VOSviewer (version 1.6.20, Leiden University, Leiden, The Netherlands), Scimago Graphica (Version 1.0.35, SRG S.L. company, Granada, Spanish), CiteSpace (version 6.3.R1, Drexel University, Philadelphia, PA, USA) and GraphPad Prism (version 10, GraphPad Prism Software Inc., San Diego, CA, USA) were used to generate tables and visual graphs. GraphPad Prism is used to generate line graphs of number of publications per year, number of citations, H-index, etc. Scimago Graphica is used to draw a map of the geographic distribution of publications. VOSviewer to create visual graphs and analyze the most productive/collaborative countries, institutions and authors, as well as the most cited journals and most co-occurrence keywords. CiteSpace for creating timeline graphs and keyword bursts. Each point on the visualization map represents a country, institution, author or journal, and these points are grouped according to their collaboration. The size of the dots depends on the number of publications. The thickness of the connecting lines connecting the nodes represents the strength of the collaboration between the nodes and the total link strength (TLS) reflects the overall level of collaboration. In the keyword analysis, some meaningless keywords were excluded and keywords with the same meaning were merged to get a better perspective. The modularity value (*Q*-value) > 0.3 and the average profile value (*S*-value) > 0.7 of the graphs generated by CiteSpace indicate that the clustering is significant and reasonable (Ling et al., 2023).

## 3 Results

### 3.1 Study selection and characteristics

**Figure 1:**
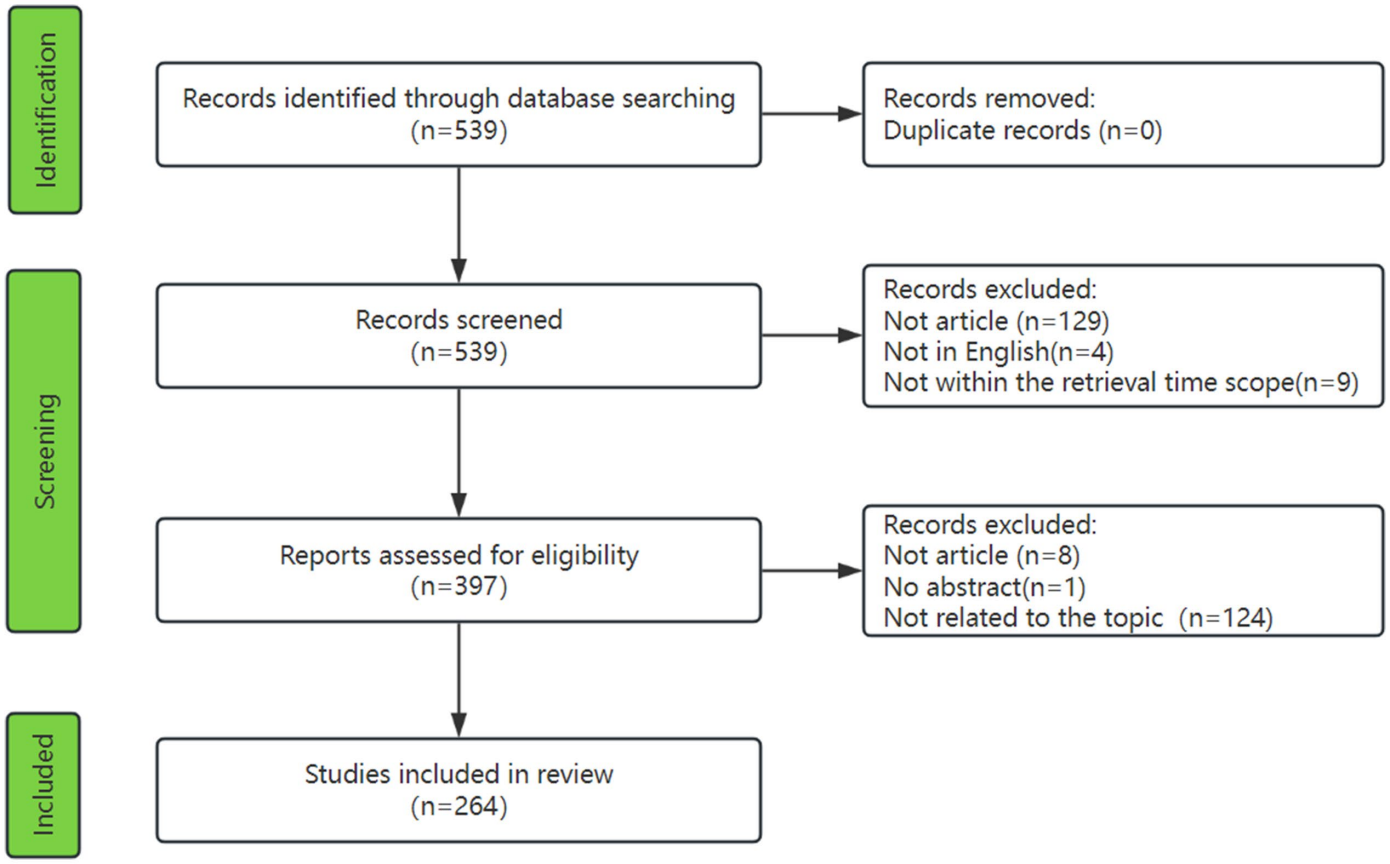
Flowchart of the literature screening process.

As shown in Figure 1, a total of 539 publications were identified from the WOSCC database by searching for keywords related to phage and *A. baumannii*, and no duplicates were found. At the first selection stage, 4 publications were excluded due to language restrictions and 129 publications were excluded due to publication types. Nine publications were excluded because they were not within the retrieval time scope. The remaining 397 publications were carefully evaluated in the second stage using article titles and abstracts. Finally, A total of 264 studies that met the inclusion criteria were selected.

### 3.2 Analysis of the annual output of publications

**Figure 2:**
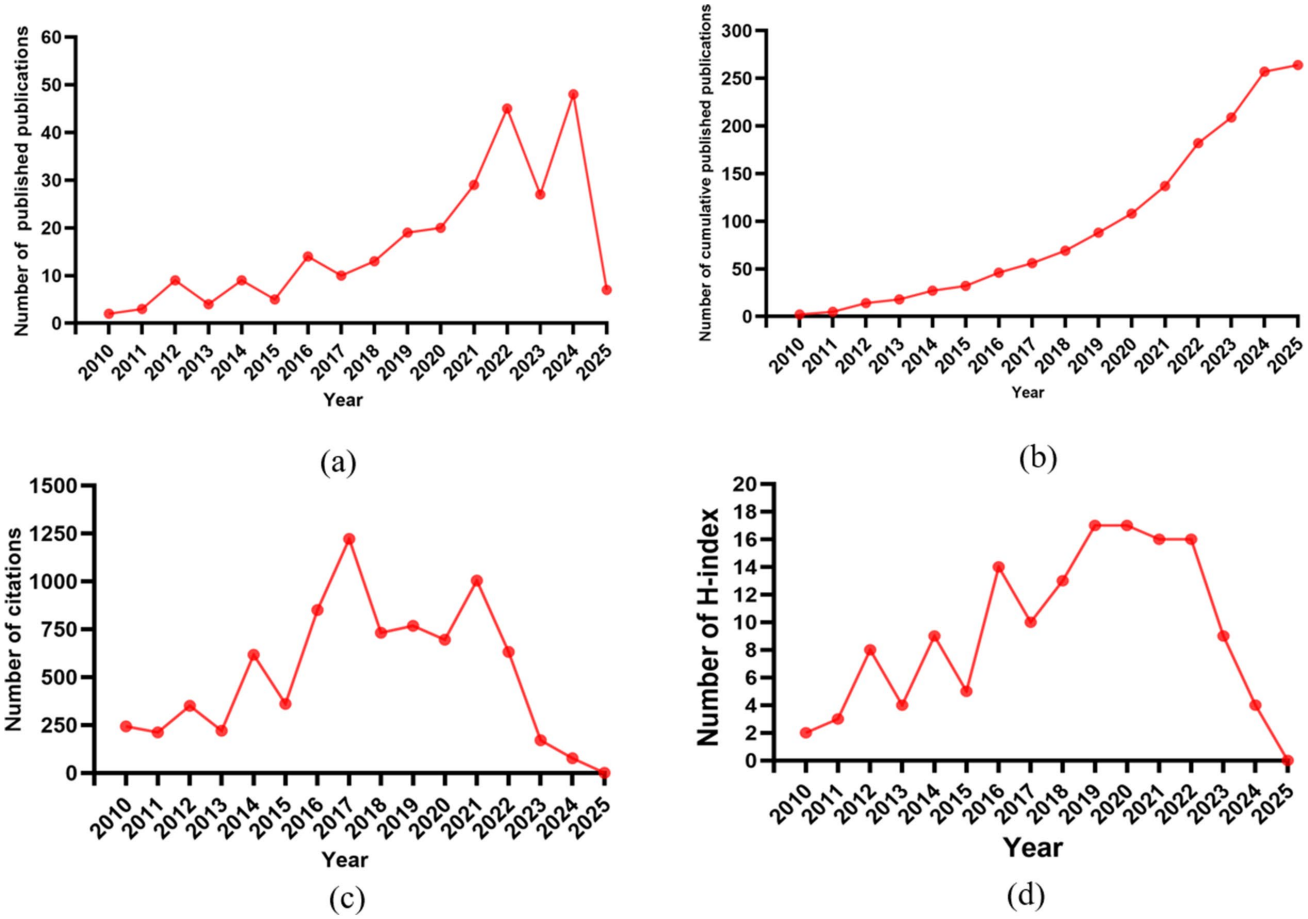
Overview of global annual publications. **(a)** The global annual number of publications. **(b)** The global annual number of cumulative publications. **(c)** The global annual number of citations of the publications. **(d)** The global annual H-index values of the publications.

The number of academic papers published each year provides insight into the past of the field and predicts its future development, and is an important indicator for visually assessing trends in scientific research. We then summarize the characteristics of the included studies. The distribution of the number of annual publications from 2010 to 2025 is shown in Figure 2a. The overall increasing trend in the number of annual publications indicates an increased interest in the field of phage and *A. baumannii* infections, peaking in 2024 with 48 publications, representing 13.18% of the total number of publications. The cumulative number of publications increased steadily from 2010 to 2025 (Figure 2b). The number of citations was relatively high from 2016 to 2021, with more than 700 citations per year (Figure 2c). The annual H-index increased from 2 in 2010 to 17 in 2019 (Figure 2d).

### 3.3 Analysis of countries/regions and institutions attributes

**Figure 3:**
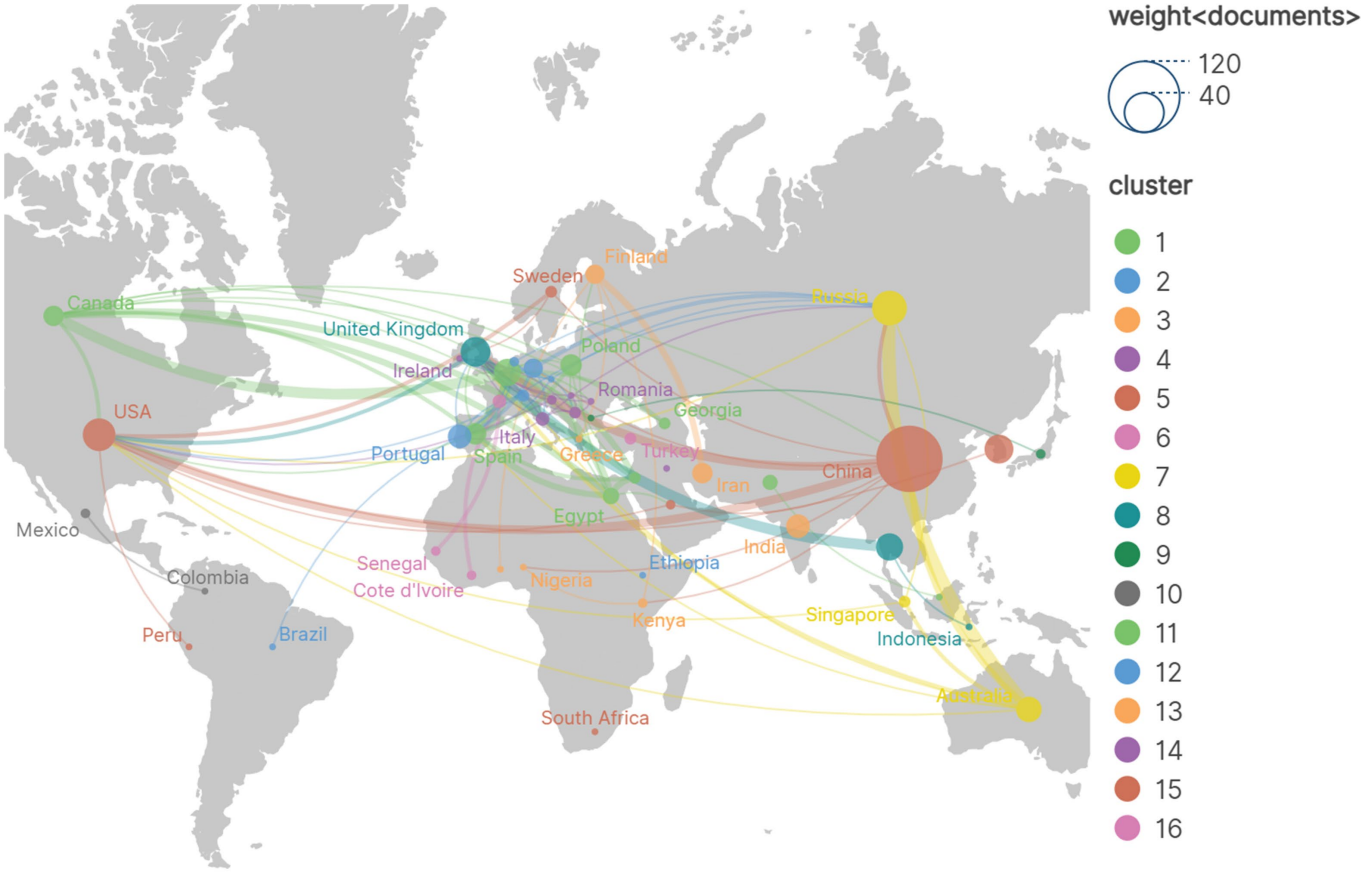
Visualization of collaboration among countries by a network map. The size of the nodes indicates the breadth of the co-operation, with thicker lines indicating a stronger relationship.

**Figure 4:**
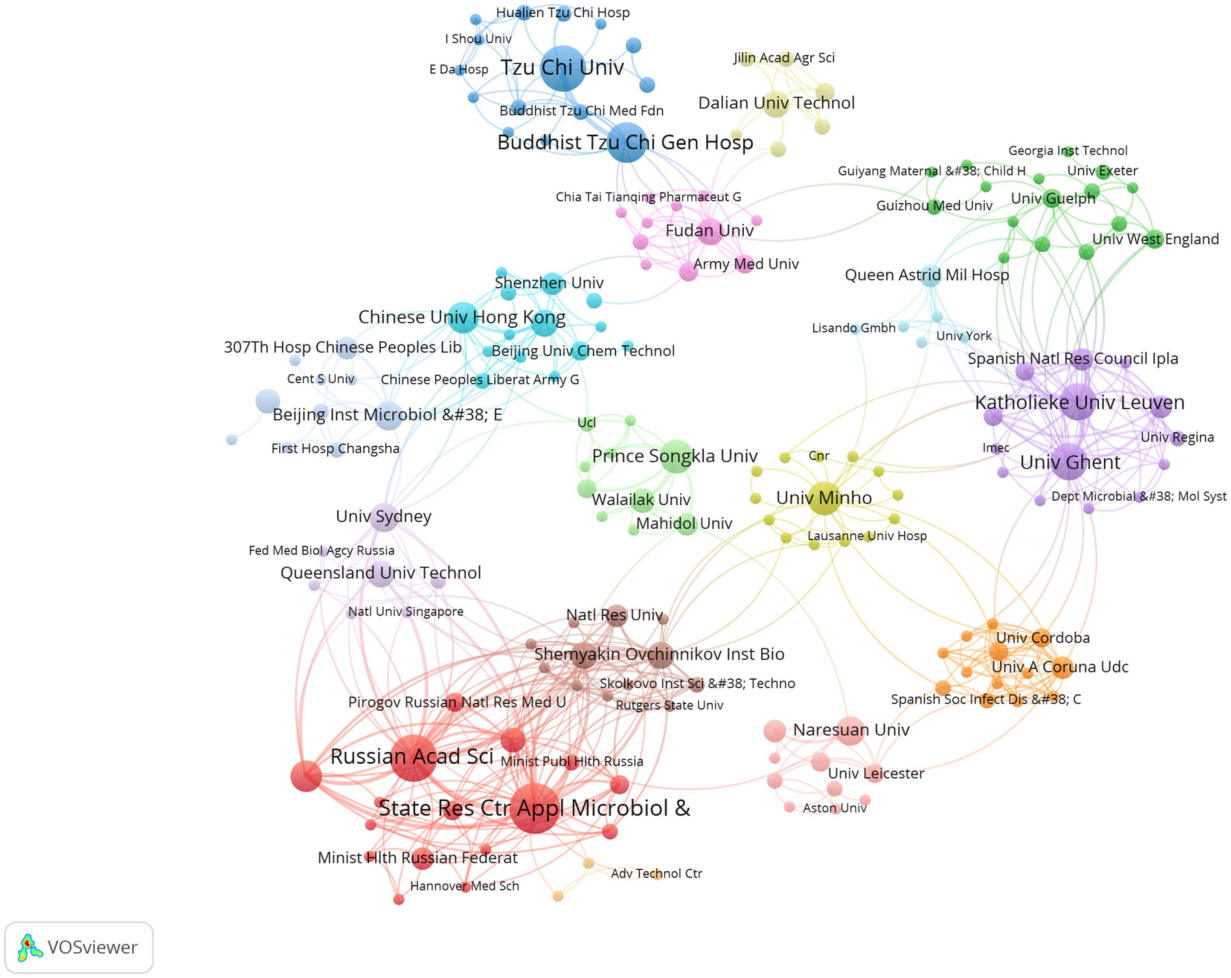
The co-authorship network map of institutions. The size of each institution’s node corresponds with its publication count. Line thickness illustrates levels of collaborative efforts among institutions in research of phages in *A. baumannii* infections.

A visualization map of the cooperation networks in each country is shown in Figure 3. A total of 51 countries/regions and 477 collaborations are shown. China has the strongest international collaboration network (TLS = 1,257) and the closest collaboration with the United States (LS = 114). We then analyze the number of publications, total citations, average citations and H-index for the 10 most productive countries. As shown in Table 1, China has the highest number of publications (95, 35.98%), followed by Russia (26, 9.85%) and the United States (23, 8.71%). China also has the highest number of citations (2686) and the highest H-index (33). The network map of institutional collaborations is shown in Figure 4, which includes 459 institutions and 13,867 collaborations. The State Research Center for Applied Microbiology and Biotechnology has the largest collaboration network (TLS = 1,640), and the largest publications (20, 7.58%), followed by the Russian Academy of Sciences (18, 6.82%) and Tzu Chi University (17, 6.44%). The 10 most productive institutions are shown in Table 2. Tzu Chi University has the highest H-index (12). Katholieke Universiteit Leuven has the highest total number of citations (841) and the highest average number of citations per paper (76.45).

**Table 1:** The top 10 most productive countries regarding phages and *A. baumannii* research from 2010 to 2025.

Rank	Country	Counts	Percentage	Total citations	Average citation per item	H-Index
1	China	95	35.98	2,686	28.27	33
2	Russia	26	9.85	496	19.08	13
3	USA	23	8.71	1750	76.09	15
4	UK	19	7.20	434	22.84	11
5	South Korea	18	6.82	435	24.17	10
6	Belgium	16	6.06	995	62.19	14
7	Thailand	16	6.06	258	16.13	10
8	Australia	14	5.30	1,267	90.50	10
9	Portugal	12	4.55	816	68.00	10
10	India	12	4.55	159	13.25	7

**Table 2:** The top 10 most productive institutions regarding phages and *A. baumannii* research from 2010 to 2025.

Rank	Institution	Counts	Percentage	Total citations	Average citations per item	H-Index
1	State Research Center for Applied Microbiology and Biotechnology	20	7.58	354	17.70	12
2	Russian Academy of Sciences	18	6.82	227	12.61	10
3	Tzu Chi University	17	6.44	723	42.53	13
4	Buddhist Tzu Chi General Hospital	13	4.92	669	51.46	10
5	Katholieke Universiteit Leuven	11	4.17	841	76.45	10
6	Ghent University	11	4.17	511	46.45	10
7	Chinese Academy of Sciences	9	3.41	184	20.44	6
8	Prince of Songkla University	9	3.41	119	13.22	6
9	University of Minho	9	3.41	665	73.89	8
10	Central Scientific Research Institute of Epidemiology	8	3.03	114	14.25	6

### 3.4 Analysis of authors of publications

**Figure 5:**
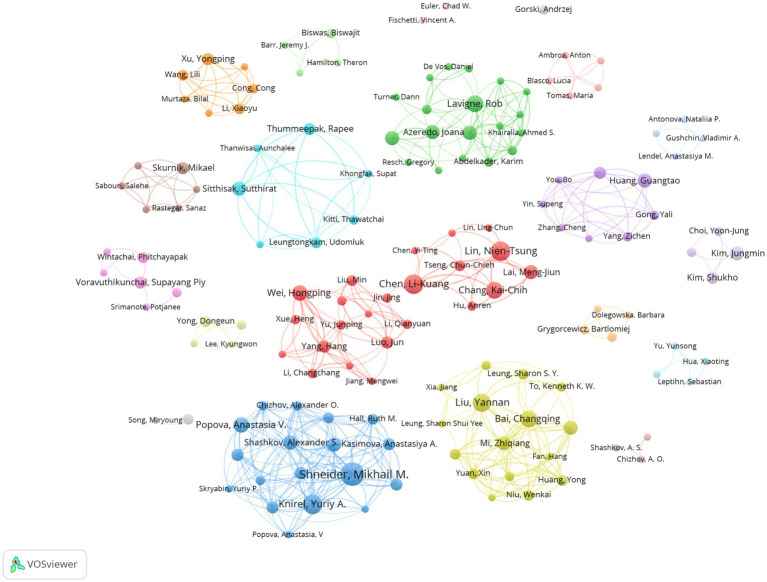
The co-authorship network map of authors. The size of each author’s node reflects their number of publications. Identical colours indicate the same cluster, and connecting lines between nodes represent collaborative links between authors.

A total of 1,503 authors were involved in all the publications analyzed. The top 10 authors with the highest number of publications are listed in Table 3. Shneider, Mikhail M. (Russia) has the highest number of publications in this field with 14 papers, followed by Knirel, Yuriy A. (Russia), Lin, Nien-Tsung (China) and Chen, Li-Kuang (China), who have all published 11 papers. Among these authors, Lin, Nien-Tsung has the highest H-index (10). In addition, Lavigne, Rob (Belgium) has the highest total number of citations and the average number of citations per paper. Shneider, Mikhail M. has the highest number of collaborations with other authors (total link strength TLS = 150). The author collaboration network diagram is shown in Figure 5. It can be found that there is strong collaboration within the teams, but fewer inter-team connections and insufficient cross-team collaboration. It is recommended to strengthen cross-team collaboration in the future to build a wider academic network.

**Table 3:** The top 10 most productive authors regarding phages and *A. baumannii* research from 2010 to 2025.

Rank	Authors	Counts	Percentage	Total citations	Average citation per item	H-Index
1	Shneider, Mikhail M.	14	5.30	159	11.36	9
2	Lin, Nien-Tsung	11	4.17	612	55.64	10
3	Knirel, Yuriy A.	11	4.17	102	9.27	7
4	Chen, Li-Kuang	11	4.17	610	55.45	9
5	Liu, Yannan	10	3.79	250	25.00	9
6	Lavigne, Rob	9	3.41	740	82.22	8
7	Chang, Kai-Chih	9	3.41	528	58.67	8
8	Bai, Changqing	9	3.41	291	32.33	9
9	Popova, Anastasia V.	8	3.03	142	17.75	5
10	Wei, Hongping	8	3.03	106	13.25	5

### 3.5 Analysis of source journals

**Figure 6:**
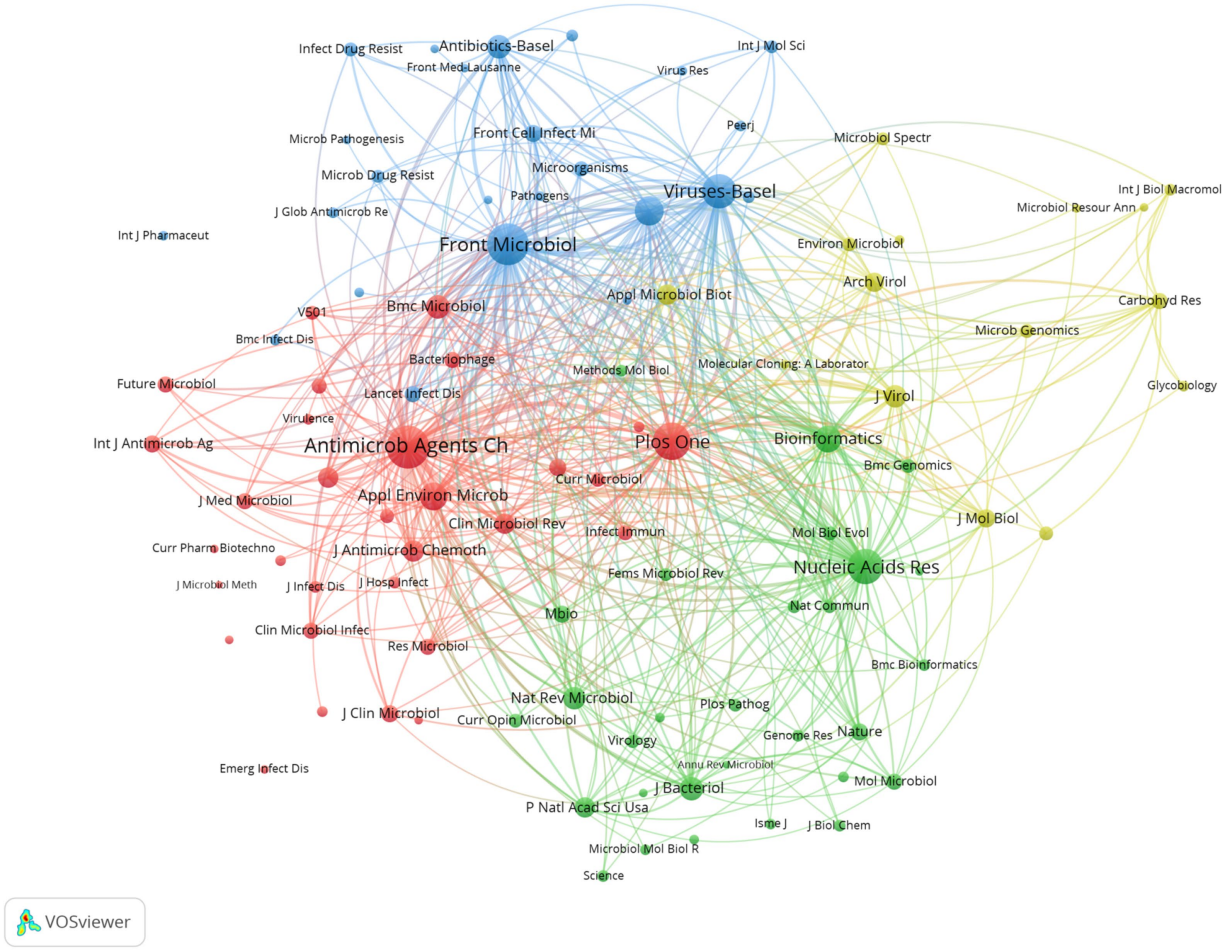
The co-authorship network map of journals. The size of each journal node reflects the journal’s activity. Identical colours indicate clustering in common subject areas, and the thickness of the connecting lines between nodes reflects the strength of co-authorship between journals.

A total of 103 academic journals were active in the field of phage in *A. baumannii* infections. The top 10 journals published 116 articles (Table 4), accounting for 43.94% of all publications. Viruses-Basel, Frontiers in Microbiology and Archives of Virology were the top three journals publishing research in this area. Antimicrobial Agents and Chemotherapy had the highest number of citations and the highest average number of citations per article. Frontiers in Microbiology has the highest H-index (18), followed by Viruses-Basel (13). A network diagram of the most cited journals in this field is shown in Figure 6.

**Table 4:** The top 10 most productive journals regarding phages and *A. baumannii* research from 2010 to 2025.

Rank	Journal title	Records	Total citations	Average citation per item	H-Index	IF(2024)
1	Viruses-Basel	24	399	16.63	13	3.8
2	Frontiers in Microbiology	23	945	41.09	18	4.0
3	Archives of Virology	12	152	12.67	7	2.5
4	Scientific Reports	12	409	34.08	10	3.8
5	Antimicrobial Agents and Chemotherapy	10	1,397	139.70	9	4.1
6	PLoS One	10	473	47.30	9	2.9
7	Antibiotics-Basel	7	126	18.00	5	4.3
8	BMC Microbiology	6	388	64.67	5	4.0
9	Journal of Virology	6	185	30.83	4	4.0
10	Microbiology Spectrum	6	56	9.33	4	3.7

In addition, the impact factor (IF) of a journal is an important parameter for assessing the value of the publications it contains (Wu H. et al., 2021). Among these, ACS Nano has the highest impact factor of 4.1, followed by Frontiers in Microbiology, BMC Microbiology and Journal of Virology, all of which have a current impact factor of 4.0. The top three most cited journals, in order, are Antimicrobial Agents and Chemotherapy (1,397 citations), Frontiers in Microbiology (945 citations) and PLoS One (473 citations).

### 3.6 Analysis of highly cited studies

The 10 most cited studies during the study period are shown in Table 5. Four of these studies were conducted independently by universities in China and Australia. The remaining 6 studies were from multi-institutional collaborations. Specifically, a study published in 2017 in Antimicrobial Agents and Chemotherapy entitled ‘Development and Use of Personalized Bacteriophage-Based Therapeutic Cocktails to Treat a Patient with a Disseminated Resistant *Acinetobacter baumannii* Infection’ was cited 792 times, making it the most cited publication in the field.

**Table 5:** The top 10 most productive studies regarding phages and *A. baumannii* research from 2010 to 2025.

Rank	Title	Institution	Authors	Journal	Citations
1	Development and Use of Personalized Bacteriophage-Based Therapeutic Cocktails To Treat a Patient with a Disseminated Resistant *Acinetobacter baumannii* Infection	University of California, USA; Naval Medical Research Center, USA; Henry M. Jackson Foundation, USA; Texas A&M University, USA; San Diego State University, USA; Monash University, Australia; AmpliPhi Biosciences, USA	Schooley RT, Biswas B, Gill JJ, et al.	Antimicrobial Agents and Chemotherapy	792
2	Engineered endolysin based “Artilysins” to combat multidrug-resistant gram-negative pathogens	KU Leuven, Belgium; University of Minho, Portugal; Queen Astrid Military Hospital, Belgium; Lisando GmbH, Germany	Briers Y, Walmagh M, Van Puyenbroeck V, et al.	mBio	290
3	Novel phage lysin capable of killing the multidrug-resistant gram-negative bacterium *Acinetobacter baumannii* in a mouse bacteremia model	The Rockefeller University, USA; Centro de Investigaciones Biológicas, Spain; Hunter College, USA	Lood R, Winer BY, Pelzek AJ, et al.	Antimicrobial Agents and Chemotherapy	196
4	Bacteriophage-resistant *Acinetobacter baumannii* are resensitized to antimicrobials	Monash University, Australia	Gordillo Altamirano F, Forsyth JH, Patwa R, et al.	Nature Microbiology	185
5	Phage Therapy for a Multidrug-Resistant *Acinetobacter baumannii* Craniectomy Site Infection	University of California, USA; Naval Medical Research Center-Frederick, USA	LaVergne S, Hamilton T, Biswas B, et al.	Open Forum Infectious Diseases	170
6	Antibacterial activity of *Acinetobacter baumannii* phage ϕAB2 endolysin (LysAB2) against both gram-positive and gram-negative bacteria	Tzu Chi University, China	Lai MJ, Lin NT, Hu A, et al.	Applied Microbiology and Biotechnology	160
7	Isolation and characterization of a virulent bacteriophage AB1 of *Acinetobacter baumannii*	Tianjin University of Science and Technology, China	Yang H, Liang L, Lin S, et al.	BMC Microbiology	132
8	Structural and Enzymatic Characterization of ABgp46, a Novel Phage Endolysin with Broad Anti-Gram-Negative Bacterial Activity	University of Minho, Portugal; University of Sheffield, UK; KU Leuven, Belgium; Consiglio Nazionale delle Ricerche, Italy	Oliveira H, Vilas Boas D, Mesnage S, et al.	Frontiers in Microbiology	115
9	Phage Therapy as a Promising New Treatment for Lung Infection Caused by Carbapenem-Resistant *Acinetobacter baumannii* in Mice	Zhejiang University of Technology, China; Shanghai Jiao Tong University, China; Fudan University, China	Hua Y, Luo T, Yang Y, et al.	Frontiers in Microbiology	115
10	Isolation and characterization of phi AB2: a novel bacteriophage of *Acinetobacter baumannii*	Tzu Chi University, China	Lin NT, Chiou PY, Chang KC, et al.	Research in Microbiology	111

### 3.7 Keyword analysis of research hotspots

**Figure 7:**
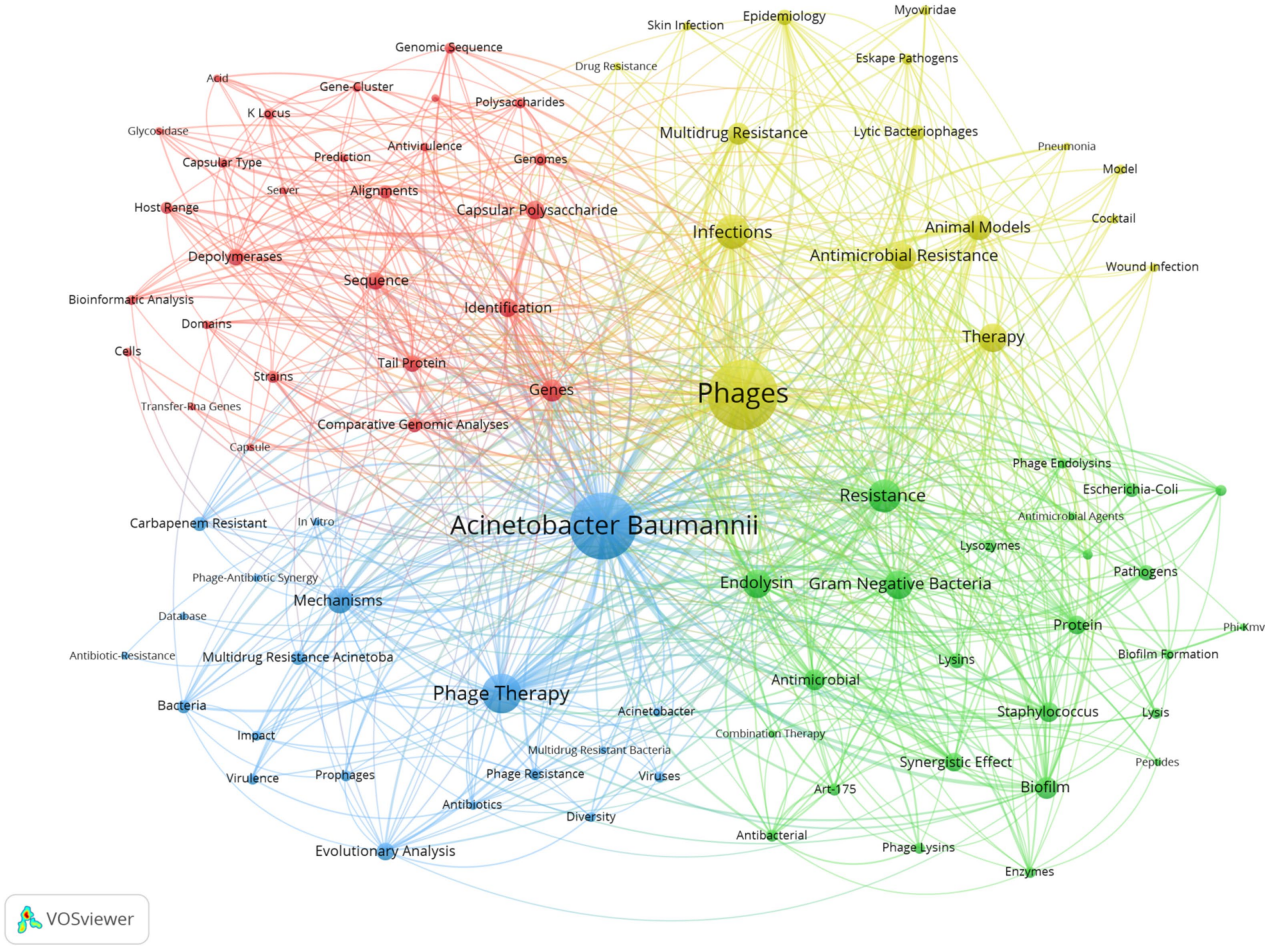
The network visualization of keywords Cooccurrence network. The size of each node indicates the size of the co-occurrence frequency of the corresponding keyword, the larger the node and font, the higher the frequency of occurrence. The thickness of the line between the nodes indicates the number of co-occurrences, and nodes of the same colour indicate the same cluster.

**Figure 8:**
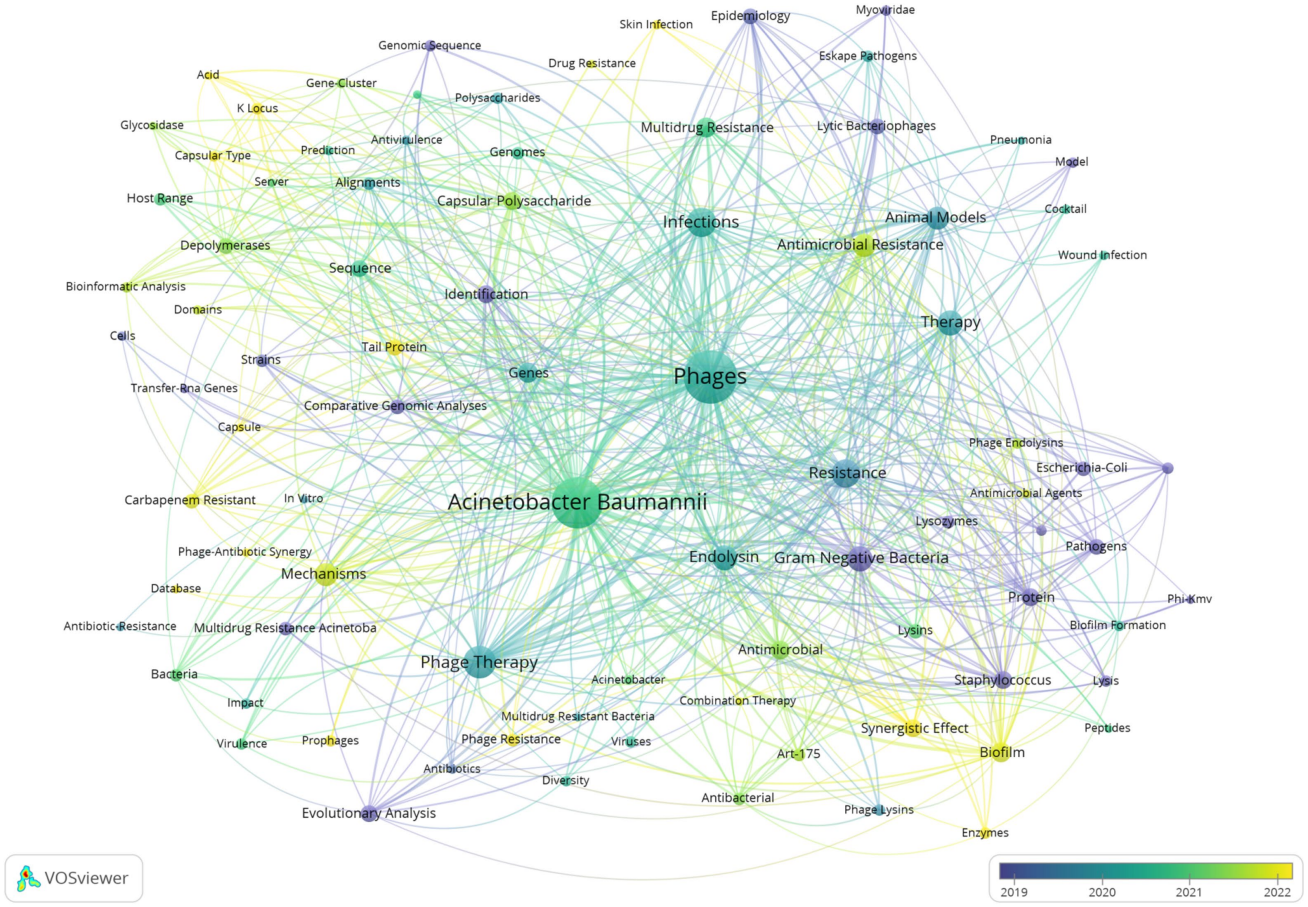
The overlay visualization of keywords Cooccurrence network. The overlay visualization shows the hotspots for phage applications in *A. baumannii* infections.

Keyword co-occurrence analysis is a common method for identifying popular research topics. The network of co-occurring keywords is shown in Figure 7. The 15 most frequent keywords were phages *A. baumannii* phage therapy infections resistance endolysin gram negative bacteria therapy antimicrobial resistance mechanisms animal models biofilm multidrug resistance genes and antimicrobial. Figure 7 shows the keywords divided into 4 clusters. The first cluster in red is related to genomics and bioinformatics analysis and included keywords such as “bioinformatic analysis” “comparative genomic analyses” “genomic sequence” “prediction” and “identification.” The second group in green is related to the study of biological structure and antimicrobial activity included keywords such as “antibacterial activity” “biofilm formation” “endolysin” “lysins” and “enzymes.” The third group in blue is related to bacterial drug resistance and phage therapy research included keywords such as “antibiotic resistance” “multidrug resistance” “phage resistance” “phage therapy” “phage-antibiotic synergy” and “prophages.” The fourth group in yellow represents infection models and antimicrobial treatment strategies included keywords such as “animal models” “pneumonia model” “epidemiology” “cocktail” and “therapy.” As shown in Figure 8 terms marked in purple indicate that their average year of publication was 2019 or earlier while those marked in bright yellow appeared after 2022. Keywords such as “pathogens” “evolutionary analysis” “multidrug resistance” and “comparative genomic analyses” were the main topics during the early stage. The keywords “biofilm” “enzymes” “synergistic” and “mechanism” appeared relatively late in the study period

**Figure 9:**
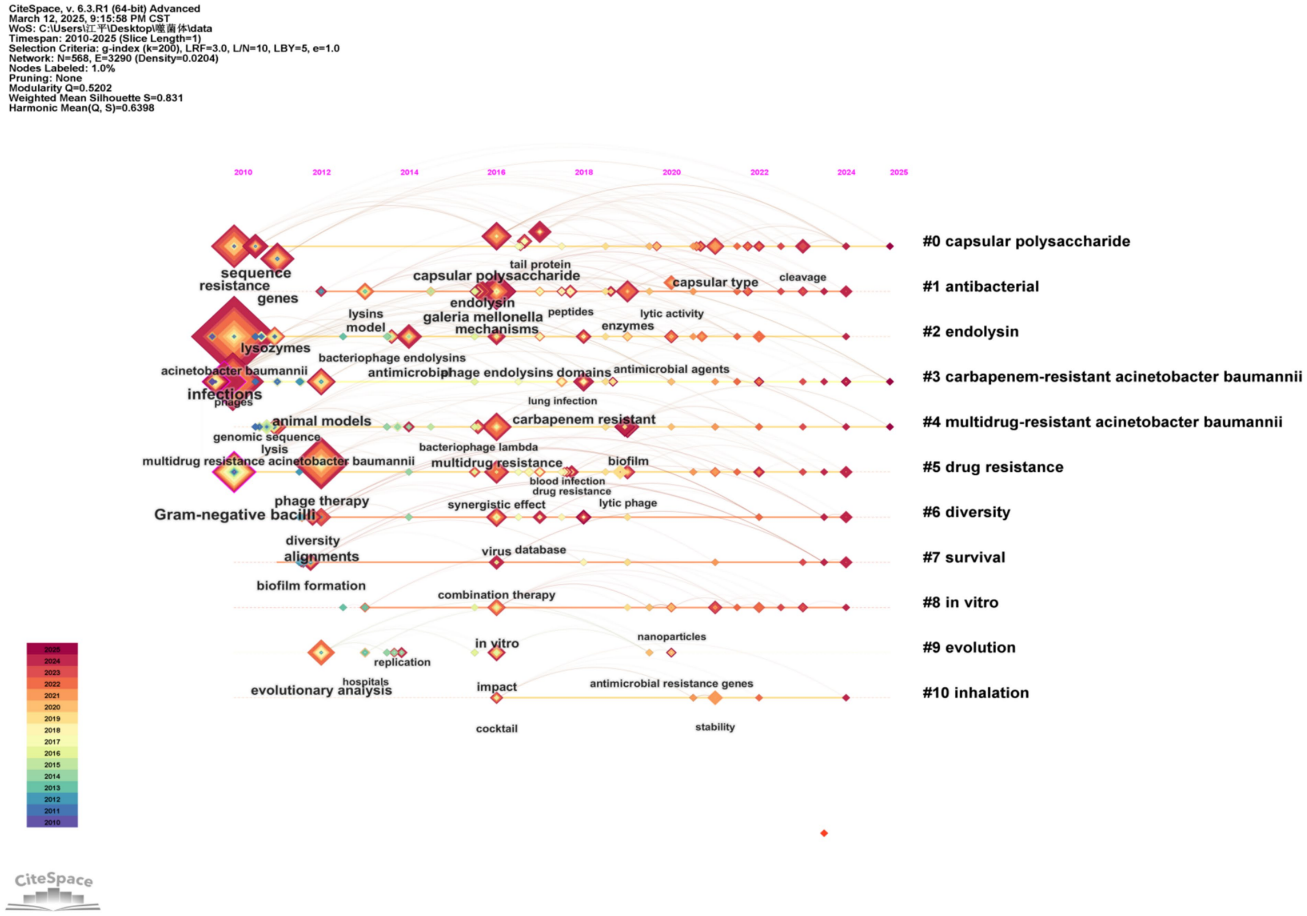
The timeline map of keywords cooccurrence. Each cluster is labelled according to the year it first appeared and different research topics are represented by colored lines.

We used CiteSpace to show a visualization of the evolution of the keywords over time (Figure 9). In this figure, the *Q* value is 0.5202 and the S value is 0.831. Recent research has indicated that the issue of drug resistance has been a central focus, especially in relation to carbapenem-resistant *A. baumannii* and multidrug-resistant *A. baumannii*. The evolutionary trajectory of research keywords over the past decade or so has demonstrated this. Phage therapy has also been a popular research topic. In recent years, research has focused on analyzing biofilm properties, searching for sequence resistance genes, and exploring the molecular mechanisms of bacterial drug resistance through *in vitro* experiments and animal models. Concurrently, researchers are developing and applying novel antimicrobial strategies such as endolysin, cocktail therapy, and phage-antibiotic synergistic effect, as well as conducting evolutionary analysis. These research progresses show that phage is developing in the direction of multidisciplinary and multi-strategy in the study of *A. baumannii* infection.

**Figure 10:**
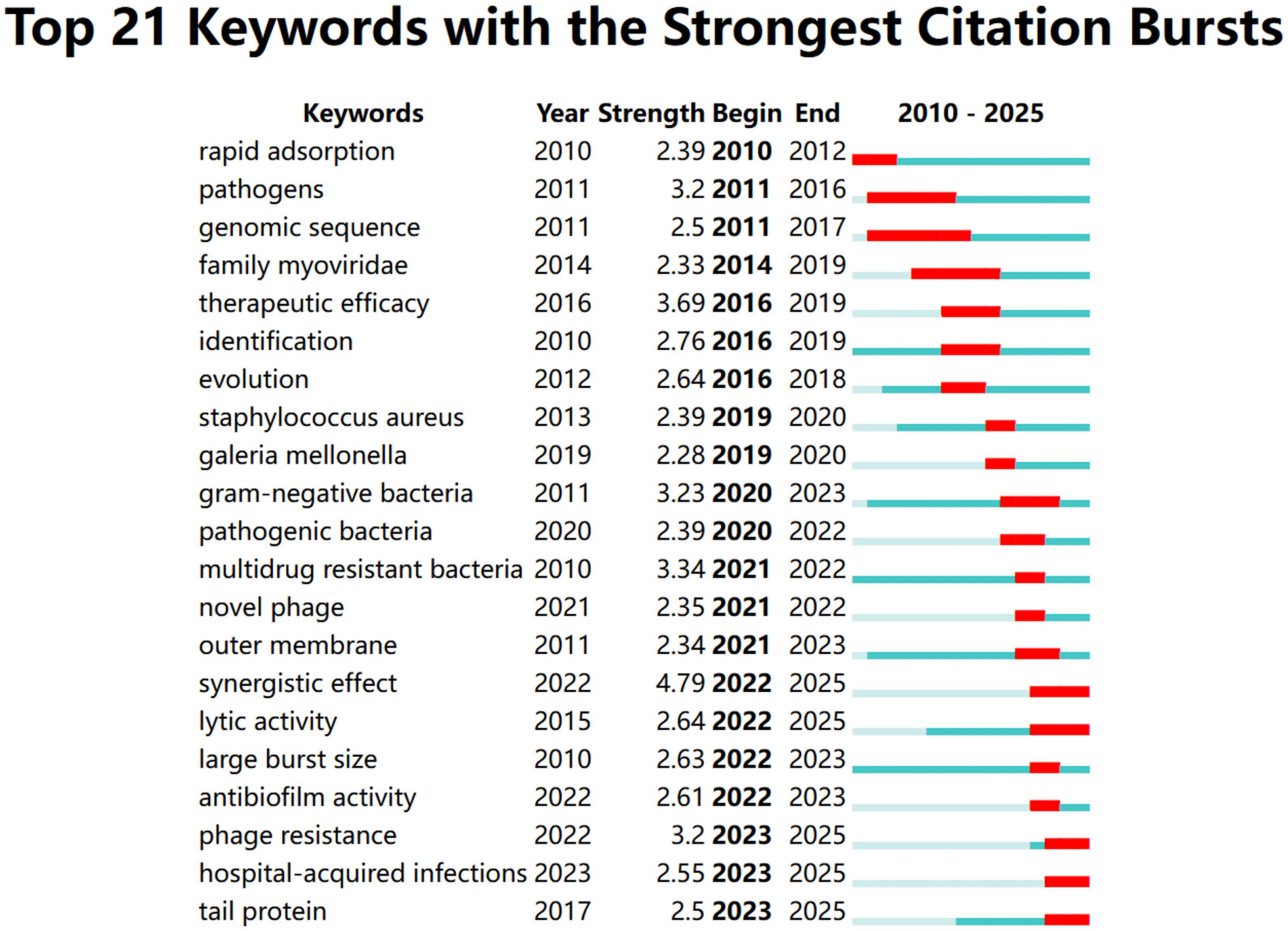
Top 21 keywords with the strongest citation bursts. A light blue line indicates a time period of frequency, a blue line indicates a time period of slightly higher frequency, and a red marker indicates a time period when the keyword’s frequency is in burst. ‘Strength’ indicates the intensity of the keyword’s citation burst, the higher the value, the higher the frequency of that time period.

Another important marker of research frontiers and hotspots throughout time is the intensity of the keyword bursts (Figure 10). Of the top 21 keywords with the strongest citation outbreaks, synergistic effect, which appeared in 2022, had the highest intensity of bursts (5.59), followed by therapeutic efficacy, which began in 2016 (3.69), and multidrug resistant bacteria, which started in 2010 (3.34). The different time periods of citation bursts for different keywords reflect the changes in research hotspots in the scientific community. The figure shows that the research hotspots have gradually shifted from “pathogens” and “genomic sequence” in the early stage to “synergistic effect”, “novel phage” and “antibiofilm activity” in the later stage. This shift may reflect the interest of the scientific community in emerging issues. It is noteworthy that the keywords “rapid adsorption,” “pathogens,” “genetic sequence,” “gram-negative bacteria,” “identification,” and “multidrug resistant bacteria” show continued research interest, indicating that these are still popular topics. In addition, based on the end years of the keyword citation bursts shown in the Figure 10, it can be predicted that the hotspots that will continue to receive attention in the next few years may be “phage resistance,” “synergistic effect” and “hospital-acquired infections.”

## 4 Discussion

To the best of our knowledge, this is the first bibliometric analysis of the literature related to the use of phages in *A. baumannii* infections. Considering the sparse number of relevant studies prior to 2010, and the fact that these studies were scattered and spanned a wide range of time, even one of them lacked abstract, it was therefore not appropriate to include these studies in this review. Consequently, for the present study, 264 studies created between 1 January 2010 and 22 February 2025, that met the inclusion criteria were selected and analyzed by multiple software tools. This analysis generated tables and visual graphs to reveal the current state of research, popular research topics and cutting-edge trends in the field.

Overall, the number of annual publications in the field fluctuates back and forth, but the general trend is one of gradual increase. The number of publications peaks in 2024. The year 2019 was an important year for the field as it had the highest H-index and has remained high in subsequent years. We then analyzed the most influential countries, institutions, authors and journals in the field. China was found to be the leader in the field, with 3.65 times more publications than the second-ranked country, which is reflected in the fact that China has the highest number of international partnerships, publications, citations, and the highest H-index, with 4 of the 10 most cited studies coming from China. In addition, Tzu Chi University and Lin, Nien-Tsung are the most influential institutions and authors in this field in terms of H-index. In addition, Frontiers in Microbiology, Viruses-Basel, and Scientific Reports are considered to be the top three most influential journals in this field. Frontiers in Microbiology has the highest H-index, while Viruses-Basel has the highest number of publications. In terms of citations, Antimicrobial Agents and Chemotherapy has the highest total number of citations and the highest average number of citations per paper.

The reason for leading in research output, especially at the national level, in the case of China, the significant share of publication may also be related to the relevant policy promotion, in addition to the necessary resource input. In 2022, China’s National Health and Health Commission formulated the National Action Plan for Containment of Microbial Resistance (2022–2025), which is a follow-up to the implementation of the previous version of the plan (2016–2020) (Yuetian and Wenjuan, 2024), it can be affirmed that China has achieved some success in AMR, such as the 2023 version of the published Guidelines for the Diagnosis, Treatment, Prevention and Control of Carbapenem-Resistant Gram-Negative Bacterial Infections (Zeng et al., 2023), but the overall resistance situation is still not optimistic. 2022 China antimicrobial resistance surveillance system (CARSS) bacterial drug resistance surveillance report (Yuetian and Wenjuan, 2024) showed that the detection rate of CRAB was as high as 53.4%. In the same year, the Centers for Disease Control and Prevention (CDC, United States) published a report on the impact of novel coronavirus infections on AMR, which indicated a significant increase in the incidence of infections caused by healthcare-associated drug-resistant pathogens, with a 78% increase in the detection rate of Centers for Disease Control and Prevention (2023), therefore, it is imperative to strengthen the scientific research and cooperation on microbial resistance prevention and control in the world.

We then analyzed the top 10 most cited studies in the field. Highly cited studies are usually considered to be important and influential. One of the most cited publications reported a clinical case study of phage therapy for drug-resistant *A. baumannii* infections (Schooley et al., 2017), successfully applied personalized phage therapy to effectively eradicate drug-resistant *A. baumannii* infections by intravenous and local injection of a phage cocktail. Another similar clinical case study among these 10 studies (LaVergne et al., 2018), which reported the case of a patient with multidrug-resistant *A. baumannii* infections treated with intravenous phage therapy after craniectomy. Although the patient did not recover from the injury, phage therapy was well tolerated with no significant adverse effects. The therapeutic experience with phages is encouraging, good clinical outcomes have been observed and the safety profile appears to be positive, but there are still few clinical trials of phage therapy for *A. baumannii* infections. There is clearly an urgent need for more clinical trials to confirm the value of phage therapy on the basis of evidence-based medicine.

In addition, 4 studies characterized the antimicrobial properties of different phages. One of them (Gordillo Altamirano et al., 2021) characterized phages ΦFG02 and ΦCO01. To exert their antimicrobial effects by targeting the capsule of *A. baumannii*, and the resistant mutant strains were re-susceptible to various antimicrobial agents due to the absence of the capsule, and the phage therapies showed significant antimicrobial effects in a mouse model. Researchers in another study (Yang et al., 2010) isolated and characterized the phage AB1, a potent phage of *A. baumannii*, which exhibited rapid growth and high pH stability and high thermal stability. The third study (Lin et al., 2010) characterized the virulent *A. baumannii* phage fAB2 isolated from hospital wastewater for the first time. fAB2 possessed broad-spectrum lysogenic activity against multidrug-resistant *A. baumannii* with the advantages of rapid adsorption, short latency and high lysogenicity. And the authors in last one study (Hua et al., 2017) isolated a phage, SH-Ab15519, showed good therapeutic efficacy against carbapenem-resistant *A. baumannii* lung infection in a mouse model, significantly improving the survival rate of the mice without obvious side effects.

Three other studies characterized the antimicrobial properties of different endolysins (lysins). Lysins are phage-encoded enzymes that degrade the bacterial cell wall at the end of the phage replication cycle, releasing newly assembled phages. They are not as specific as phages, and a single phage-encoded endolysin may be able to lysate a wide range of bacteria. One study (Lood et al., 2015) identified the novel endolysin PlyF307 showed strong bactericidal activity against multidrug-resistant *A. baumannii*, which not only efficiently cleared planktonic bacteria and biofilm *in vitro*, but also improved the survival rate of infected mice in a mouse bacteraemia model. One study (Lai et al., 2011) isolated endolysins LysAB2 from *A. baumannii* phage 6AB2, which exhibited broad-spectrum antibacterial activity against a variety of drug-resistant bacteria, including *A. baumannii*, by enhancing the permeability of bacterial cell membranes, and possessed good thermal stability. The other study (Oliveira et al., 2016) identified the novel endolysins ABgp46 from the *A. baumannii* phage vb_AbaP_CEB1 as having a broad-spectrum of antimicrobial activity against drug-resistant Gram-negative bacteria, and the antimicrobial effect was significantly enhanced when combined with citric and malic acids.

In the last study (Briers et al., 2014), artilysins developed through protein engineering successfully breached the outer membrane barrier of Gram-negative bacteria, including *A. baumannii*, and demonstrated effective bactericidal activity against drug-resistant strains. As we know, the popularity and application of sequencing technology has enabled researchers to discover many new phages (Blasco et al., 2022; Kyriakidis et al., 2021). Further analysis of the unknown functional proteins of *A. baumannii* phages will facilitate the development of phage-derived enzyme preparations. While second-generation sequencing and gene editing technologies have accelerated the screening and engineering of phages. There is still great potential in this field, despite the limited research on engineered phages that effectively degrade multidrug-resistant *A. baumannii* (Popova et al., 2019; Shchurova et al., 2021; Vukotic et al., 2020).

It is worth noting that “biofilm” is also one of the high-frequency keywords. Biofilms are composed of bacterial communities encapsulated in a fibrin matrix that impedes antibiotic penetration and enhances environmental adaptation (Leptihn and Loh, 2022). In recent years, the ability to form biofilms has been identified as a central factor in drug resistance and nosocomial infection outbreaks in *A. baumannii*. It has been shown that phage action on biofilms can disrupt the biofilm matrix, expose bacterial surface receptors and initiate the infection cycle with enhanced antibiotic penetration, resulting in a synergistic bactericidal effect (Hernandez-Morales et al., 2018). Therefore, the study of phages acting on biofilms is particularly necessary. As phages continue to be discovered and characterized to lyse biofilm-associated proteins, many studies have reported the effective elimination of *A. baumannii* using phage haemolysins or depolymerases (Huang et al., 2014; Oliveira et al., 2016), and although such proteins are unable to replicate themselves, the lower risk of resistance and the significant effect of combination with antibiotics provide new ideas for clinical treatment.

Specifically, phages can specifically lyse CRAB that are ineffective for antibiotics and are suitable for critical infections (Schooley et al., 2017). In addition, by combining with antibiotics, phages can simultaneously improve antibiotic penetration and reduce the occurrence of drug resistance when disrupting biofilms, and the synergistic effect of the two to enhance efficacy can break through biofilm-associated infections (Chan et al., 2016) The bactericidal mechanism of lytic enzymes secreted by phage is different from that of phage itself and antibiotics, and phage enzymes can dissolve more pathogenic bacteria and do not generate resistance, and phage enzyme-related agents may be widely used for biocontrol in the future (Coleman et al., 2012; Lu and Collins, 2007). Phage cocktail therapy, on the other hand, involves screening of matching phages based on strains isolated from patients to achieve personalized treatment and reduce the risk of drug resistance through phage combination therapy (Merabishvili et al., 2014).

However, phage therapy still faces several challenges. These include the limited spectrum of phage lysis and the development of bacterial resistance through rapid mutation of phage receptors (Wang et al., 2023). However, it is encouraging to note that phage cocktail therapy or phage combination therapy may be an effective response strategy. Phage cocktails have been identified as the best solution for clinical applications, but more cocktail combination therapies still need to be explored to identify the most effective cocktails and further elucidate the combination mechanisms (Caflisch et al., 2019; Cui et al., 2019; Grygorcewicz et al., 2021; Luong et al., 2020a). Combinations of phage and other antimicrobial substances are also promising for the treatment of *A. baumannii*, including phage-antibiotic combination therapies (Grygorcewicz et al., 2020), phage-natural antimicrobial combination therapies (Wintachai and Voravuthikunchai, 2022), and even phage-photosensitizer combination (Ran et al., 2020). However, further experimental studies are needed to determine the optimal combination regimen and application strategy (Gordillo Altamirano et al., 2022; Grygorcewicz et al., 2021).

More seriously, the high host specificity of phages makes it challenging to prepare phage cocktails in advance, and current cocktail therapies still require the implementation of highly individualized regimens, which leads to high investment in screening specific phages and subsequent treatment (Mattila et al., 2015). In addition, because of the widespread lack of a theoretical mechanism system for phage therapy, the current lack of authoritative guidelines or consensus on clinical application, and the possible risk of endotoxin release in the treatment of Gram-negative drug-resistant bacteria, many patients do not agree to be treated with phages. This is one of the major reasons why phage therapy has not been approved as clinical drug therapy (Knoll and Mylonakis, 2014; Schooley et al., 2017).

The rapid emergence of bacterial resistance to phages makes the application of phage therapy crucial. Therefore, there is a need for basic research, including the characterization of phage host receptors and the molecular mechanism of phage resistance in *A. baumannii.* In the future, phage therapy needs to promote the establishment of a global phage resource sharing platform based on the analysis of bacterial phage interaction mechanisms. Through the integration of precise targeting, synergistic antimicrobial agents and technological innovation, phage therapy is expected to break the multidrug resistance dilemma and open a new avenue for anti-infection treatment.

This study also has some limitations. Firstly, the literature was only retrieved from the WOSCC database, which may have resulted in an incomplete search. However, this database is one of the largest and most comprehensive databases in the world and is the most commonly used source of literature in bibliometric analyses, and the size of its data is sufficient to reflect the current state of research in the field. Secondly, we only selected studies published in English. Finally, some citation metrics in bibliometric analyses may be somewhat biased because citations are affected by various factors, such as time windows, self citation, author authority, journal impact and open access status (Urlings et al., 2021). For example, because of the lag in citations, the number of cited studies in recent years is relatively small.

## 5 Conclusion

In summary, we performed bibliometric analyses using various statistical software to obtain an overview on phages in *A. baumannii* infections. We enumerate the characteristics of publications, show the most influential countries, institutions, authors, and journals, and visual the research hotspots in the field of phage therapy along with their application for the prevention and treatment of *A. baumannii* infections. In addition, we discuss the advantages and challenges faced by phages in the control of *A. baumannii* infections. Phage therapy can be a powerful tool for the treatment of *A. baumannii* infections.

## Data Availability

The original contributions presented in the study are included in the article/Supplementary material, further inquiries can be directed to the corresponding author/s.
